# Diverse Responses in Lattice Thermal Conductivity of *n*‐Type/*p*‐Type Wurtzite Semiconductors Driven by Asymmetric Electron‐Phonon Interactions

**DOI:** 10.1002/advs.202514910

**Published:** 2025-10-27

**Authors:** Jianshi Sun, Shouhang Li, Zhen Tong, Cheng Shao, Han Xie, Meng An, Chuang Zhang, Xiongfei Zhu, Chen Huang, Quanjie Wang, Yucheng Xiong, Xiangjun Liu

**Affiliations:** ^1^ Institute of Micro/Nano Electromechanical System and Integrated Circuit College of Mechanical Engineering Donghua University Shanghai 201620 China; ^2^ Centre de Nanosciences et de Nanotechnologies, CNRS Université Paris‐Saclay 10 Boulevard Thomas Gobert Palaiseau 91120 France; ^3^ School of Advanced Energy Sun Yat‐Sen University Shenzhen 518107 China; ^4^ Thermal Science Research Center Shandong Institute of Advanced Technology Jinan Shandong 250103 China; ^5^ School of Energy and Materials Shanghai Polytechnic University Shanghai 201209 China; ^6^ Department of Mechanical Engineering The University of Tokyo 7‐3‐1 Hongo, Bunkyo Tokyo 113‐8656 Japan; ^7^ School of Mechanical Engineering Shandong Key Laboratory of CNC Machine Tool Functional Components Qilu University of Technology (Shandong Academy of Sciences) Jinan 250353 China

**Keywords:** electron‐phonon coupling, first‐principles calculations, lattice thermal conductivity, wurtzite semiconductors

## Abstract

Accurately assessing the impact of electron‐phonon interaction (EPI) on the lattice thermal conductivity of wurtzite semiconductors is crucial for the thermal management of electronic devices, and a unified physical understanding of this issue is highly desired. In this work, the lattice thermal conductivities of typical direct and indirect bandgap wurtzite semiconductors are predicted, accounting for EPI based on mode‐level first‐principles calculations. It is found that there exist diverse responses in the lattice thermal conductivity of *n*‐type/*p*‐type wurtzite semiconductors concerning charge carrier concentrations due to asymmetric EPIs. The EPI has a larger effect on the lattice thermal conductivity of *p*‐type doping compared to *n*‐type doping in the same wurtzite semiconductor. The stronger EPI in *p*‐type doping is attributed to the relatively higher electron density of states caused by the *p*‐orbital component. Furthermore, EPI has a stronger influence on the lattice thermal conductivity of *n*‐type indirect bandgap wurtzite semiconductors than *n*‐type direct bandgap wurtzite semiconductors. This is attributed to the relatively lower electron density of states in direct bandgap wurtzite semiconductors stemming from the *s*‐orbital component.

## Graphical abstract

It is traditionally thought that the electron‐phonon interactions have a significant impact on the lattice thermal conductivity of both heavily doped *p*‐type and *n*‐type semiconductors. However, it is found that this is invalid in most wurtzite semiconductors. The diversity of lattice thermal conductivity responses to asymmetric electron‐phonon interaction arises from differences in the orbital composition of the electronic density of states.

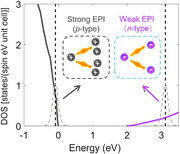

## 1 Introduction

Semiconductors serve as the cornerstone of the modern information technology industry. Charge carrier transport in semiconductors is responsible for electrical current, yet their scattering with the lattice is inevitable. The electron‐phonon interaction (EPI) has been extensively studied for its impacts on electrical transport properties, including carrier mobility, electrical thermal conductivity, and superconductivity.^[^ ^1^, ^2^, ^3^, ^4^, ^5^, ^6^, ^7^, ^8^, ^9^, ^10^, ^11^, ^12^ ^]^ However, the EPI can also affect the thermal transport properties of thermoelectric materials^[^ ^13^, ^14^, ^15^, ^16^, ^17^, ^18^ ^]^ and nano‐electronic devices^[^ ^19^, ^20^ ^]^ when the charge carrier concentrations reach as high as 10^20^ to 10^21^ cm^−3^. Therefore, it is critical to reveal the effects of EPI on the lattice thermal conductivity to accurately predict the performance and reliability of semiconductor‐based devices.^[^ ^21^ ^]^

The methodology of extracting force constants from first‐principles calculations combined with the Peierls–Boltzmann transport equation (PBTE) has been demonstrated to robustly predict the lattice thermal conductivity of intrinsic semiconductors.^[^ ^22^, ^23^, ^24^, ^25^, ^26^, ^27^, ^28^, ^29^, ^30^ ^]^ In addition, PBTE can provide abundant physical information, such as the phonon mean free path and Grüneisen parameter, which are critical to understanding the thermal transport mechanisms. The key step in solving the PBTE is to obtain various phonon scattering rates. Milestone progress has been achieved in the solution of phonon‐phonon, phonon‐isotope, phonon‐impurity, and phonon‐boundary scattering rates.^[^ ^31^ ^]^ To achieve excellent electrical transport properties in semiconductor‐based devices, doping is typically required to attain higher carrier concentrations. For instance, the electrical conductivity of *n*‐type aluminum nitride (AlN) increases by four orders of magnitude as the silicon doping concentration ranges from 1.2 × 10^21^ to 2.5 × 10^21^ cm^−3^.^[^ ^32^ ^]^ Similarly, the resistivity is reduced to 0.2 Ω·cm by increasing the concentration of implanted manganese and oxygen ion to 2 × 10^18^ cm^−3^ for *p*‐type gallium nitride (GaN).^[^ ^33^ ^]^ However, high carrier concentration inevitably introduces an additional phonon‐electron scattering term, which has long been ignored in previous empirical models for lattice thermal conductivity.^[^ ^34^ ^]^ For example, Fang et al. found that the temperature dependence of the lattice thermal conductivity of silicon at a carrier concentration of 9.3 × 10^19^ cm^−3^ can be well fitted only when the electron‐phonon scattering term is incorporated in the Debye‐Callaway model.^[^ ^35^ ^]^ Similarly, the room‐temperature lattice thermal conductivity of heavily doped Si_0_ *_._*_94_P_0_ *_._*_06_ significantly deviates from the experimental values when the electron‐phonon scattering term is ignored in the Callaway model.^[^ ^36^ ^]^ Furthermore, Wang et al. found that the temperature dependence of the lattice thermal conductivity of the 100 nm InAs nanowire matches well with the experimental data after incorporating the surface electron‐phonon scattering term in the Callaway model^[^ ^20^ ^]^.

Liao et al. found that the lattice thermal conductivity of silicon is significantly reduced by ≈37% for an electron concentration of 10^21^ cm^−3^ and by ≈45% for a hole concentration of 10^21^ cm^−3^ at room temperature.^[^ ^37^ ^]^ Subsequent experiments using ultrafast photoacoustic spectroscopy confirmed significant suppression of phonon propagation by phonon‐electron scattering in silicon at room temperature.^[^ ^38^ ^]^ Similar trends were also observed in other semiconductors such as 3C‐silicon carbide (3C‐SiC)^[^ ^39^ ^]^ and silicon‐germanium (SiGe) alloy.^[^ ^40^ ^]^ The aforementioned studies mainly focused on face‐centered cubic (*fcc*) semiconductors, where both electrons and holes are strongly coupled with phonons. Wurtzite semiconductors, like GaN, AlN, and 2H‐silicon carbide (2H‐SiC), exhibit high Baliga figure of merits, making them promising materials for power electronics.^[^ ^41^ ^]^ It was previously found that the effect of EPI on the lattice thermal conductivity of wurtzite GaN is different from that in *fcc* semiconductors. The EPI has a weak impact on the lattice thermal conductivity of *n*‐type wurtzite GaN at ultra‐high electron concentrations due to fewer phonon‐electron scattering channels.^[^ ^42^ ^]^ In contrast, there is a significant reduction in the lattice thermal conductivity of *p*‐type wurtzite GaN at high hole concentrations. The lattice thermal conductivity of wurtzite ZnO is primarily suppressed by Fröhlich interactions at low electron concentrations, whereas deformation‐potential scattering becomes the dominant limiting factor at high electron concentrations.^[^ ^43^ ^]^ Moreover, it was also found that the EPI can induce a significant reduction in the lattice thermal conductivity of 2H‐SiC within the electron concentration range of 10^17^ to 10^18^ cm^−3^ at low temperatures.^[^ ^44^ ^]^ There are more atoms in the primitive cell of wurtzite semiconductors compared to those in *fcc* structures, making their phonon dispersions and electronic band structures more complicated. There is still a lack of a unified physical understanding regarding the impact of EPI on the lattice thermal conductivities in *n*‐type and *p*‐type wurtzite semiconductors.

In this work, we investigate the EPI effects on the lattice thermal conductivity of direct/indirect bandgap wurtzite semiconductors, using rigorous mode‐level first‐principles calculations combined with the PBTE. For direct bandgap semiconductors, GaN, zinc oxide (ZnO), AlN, and gallium phosphide (GaP) are chosen, while for the indirect bandgap semiconductor, 2H‐SiC and boron nitride (BN) are chosen. We provide a comprehensive analysis of the contribution terms attributed to EPI, including electron density of state (DOS), Fermi surface nesting function, and electron‐phonon matrix element. We reveal that the diverse responses in lattice thermal conductivity of *n*‐type/*p*‐type wurtzite semiconductors stem from asymmetric EPIs.

## 2 Results and Discussion

### 2.1 Direct Bandgap Wurtzite Semiconductors

**Figure 1:**
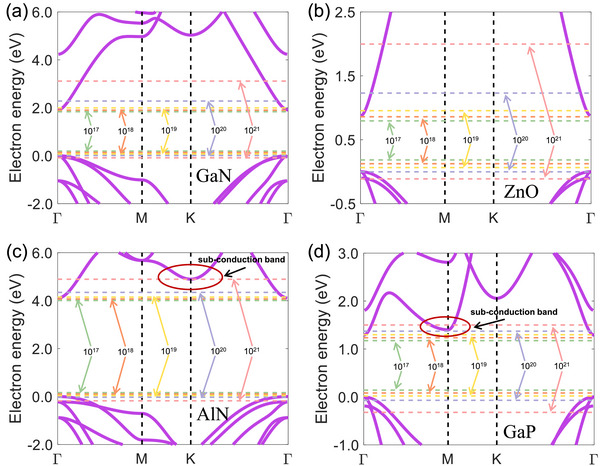
Band structures of a) GaN, b) ZnO, c) AlN, and d) GaP along the high symmetry paths, respectively. The horizontal lines are Fermi energy related to the carrier concentrations of 10^17^ (green), 10^18^ (orange), 10^19^ (yellow), 10^20^ (light purple), and 10^21^ (light pink) cm*^−^* ^3^ at room temperature. The electron energy is normalized to the VBM.

**Figure** **1** shows the electron band structures of direct bandgap wurtzite semiconductors along the high‐symmetry path Γ‐M‐K‐Γ in the first Brillouin zone. The impact of spin‐orbit coupling (SOC) on κ_lat_ is considered in this work.^[^ ^42^ ^]^ The Fermi energy corresponding to different carrier concentrations is depicted by horizontal dashed lines. The band structures of GaN, ZnO, AlN, and GaP show that both the valence band maximum (VBM) and conduction band minimum (CBM) are located at the Γ‐point, resulting in direct bandgaps of 1.86, 0.87, 4.11, and 1.29 eV, respectively. All bandgaps are significantly underestimated compared to experimental data,^[^ ^45^, ^46^, ^47^, ^48^ ^]^ attributed to the well‐known limitations of DFT.^[^ ^49^ ^]^ Nevertheless, this discrepancy does not impact our conclusions regarding the EPI effects on κ_lat_ since only electron modes near the band edges contribute to EPI, and the profiles of our DFT band structures agree well with experimental data.^[^ ^45^, ^46^, ^47^, ^48^ ^]^ It should be noted that the sub‐conduction bands of AlN and GaP are quite close to their CBM, as shown in Figure 1c,d. This phenomenon results in an anomalous decrease in κ_lat_ when the electron concentration reaches a certain level, as will be discussed later.

**Figure 2:**
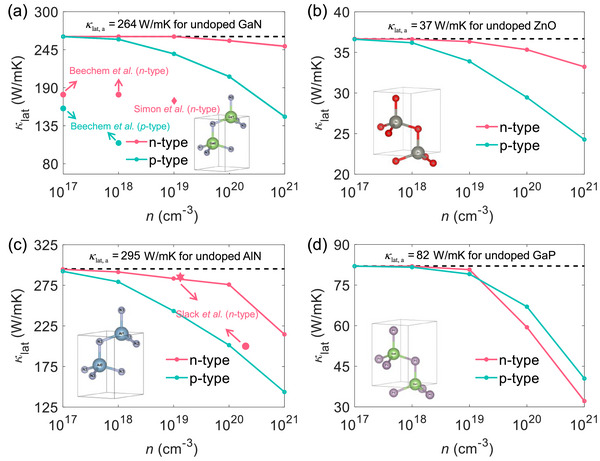
The κ_lat, *a*_ as a function of carrier concentration at room temperature for undoped, *n*‐type, and *p*‐type a) GaN, b) ZnO, c) AlN, and d) GaP, respectively. The crystal structure schematics are inserted at the bottom of the (a–d). The scatters are experimental results, reported by Beechem et al.,^[^ ^53^ ^]^ Simon et al.,^[^ ^54^ ^]^ and Slack et al.,^[^ ^55^, ^56^ ^]^ respectively.

**Figure** **2** shows the in‐plane lattice thermal conductivity (κ_lat, *a*_) as a function of carrier concentrations. The κ_lat, *a*_ at room temperature for undoped GaN and ZnO is 264 and 37 W mK^−1^, respectively. Our calculated κ_lat, *a*_ agrees well with the experimental data^[^ ^50^, ^51^ ^]^ and former theoretical results.^[^ ^42^, ^52^ ^]^ With phonon‐electron scattering further included, the κ_lat, *a*_ of *n*‐type GaN falls within the range of 250–264 W mK^−1^, which is quite close to the undoped case. In contrast, κ_lat, *a*_ dramatically decreases from 264 to 148 W mK^−1^ within the hole concentration range of 10^17^ to 10^21^ cm^−3^. A similar trend is also seen in time‐domain thermoreflectance (TDTR) measurements,^[^ ^53^, ^54^ ^]^ where the κ_lat_ of *n*‐type GaN remains nearly constant within the doping concentration range of 10^17^ to 10^19^ cm^−3^. However, the κ_lat_ of *p*‐type GaN shows a significant reduction. The quantitative differences between our work and experimental values may be attributed to impurities, defects, or experimental sample quality, while the slight disparities between our work and theoretical values may stem from the different pseudopotentials used in DFT calculations. Similar trends are also observed in ZnO. The κ_lat, *a*_ of *n*‐type ZnO only decreased by 9.4% within the electron concentration range of 10^17^ to 10^21^ cm^−3^, while the κ_lat, *a*_ of *p*‐type ZnO decreased by 33.8% within the hole concentration range of 10^17^ to 10^21^ cm^−3^. Interestingly, the variation in κ_lat, *a*_ exhibits an anomalous trend for *n*‐type AlN and GaP. For AlN, when the electron concentration locates in the range of 10^17^ to 10^20^ cm^−3^, κ_lat, *a*_ experiences a slight decrease. As the electron concentration rises from 10^20^ to 10^21^ cm^−3^, there is an abrupt 22% decrease in κ_lat, *a*_, a trend similar to the experimental data reported in Refs. [55, 56]. For GaP, the κ_lat, *a*_ exhibits a significant decline at an electron concentration of 10^20^ cm^−3^, which results in the κ_lat, *a*_ of *n*‐type GaP being lower than that of *p*‐type GaP. Overall, the EPI induced by holes has a stronger impact on κ_lat_ in direct wurtzite semiconductors compared to that induced by electrons, except for cases with sub‐conduction bands close to the CBM.

**Figure 3:**
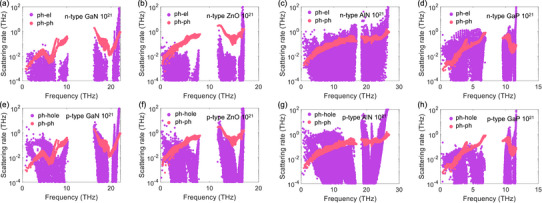
Phonon‐phonon scattering rates, a–d) phonon‐electron scattering rates, and e–h) phonon‐hole scattering rates for GaN, ZnO, AlN, and GaP at room temperature with the carrier concentration of 10^21^ cm*^−^* ^3^.

To reveal the underlying mechanisms, the mode‐level phonon‐electron and phonon‐hole scattering rates for direct bandgap wurtzite semiconductors at the carrier concentration of 10^21^ cm^−3^ are calculated, as shown in **Figure** **3**. The phonon‐electron and phonon‐hole scattering rates for high‐frequency optical phonons are relatively larger due to the Fröhlich interaction in polar materials.^[^ ^57^ ^]^ However, the contribution of high‐frequency optical phonon modes to the κ_lat_ can be neglected.^[^ ^42^, ^58^ ^]^ In the following discussions, we mainly focus on the phonon‐electron and phonon‐hole scattering rates below the phonon frequency gap since they primarily contribute to κ_lat_. For GaN and ZnO, the phonon‐electron scattering rates are significantly lower than the phonon‐phonon scattering rates, whereas the phonon‐hole scattering rates are notably higher than the phonon‐phonon scattering rates. However, both phonon‐electron and phonon‐hole scattering rates in AlN and GaP are significantly higher than the phonon‐phonon scattering rates. Specifically, the phonon‐hole scattering rate dominates over the phonon‐electron scattering rate in AlN, whereas the opposite trend is observed in GaP. In contrast, the scattering rate trend in AlN is similar to that of GaN when the carrier concentration is 10^20^ cm^−3^, as shown in [Figure S5, Supporting Information].

**Figure 4:**
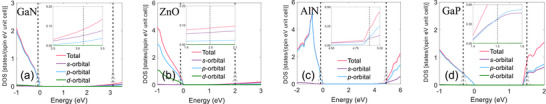
a) Total and partial DOS near the valence‐ and conduction‐band edges of (a) GaN, b) ZnO, c) AlN, and d) GaP, respectively. The gray dotted curve represents the Fermi window, indicating the relevant range of energies. The electron energy is normalized to the VBM. The position of the Fermi energy for electron and hole concentrations of 10^21^ cm*^−^* ^3^ is indicated with black dashed lines. The enlarged pictures of total and partial DOS for the Fermi energy with the electron concentration of 10^21^ cm*^−^* ^3^ are inserted above in (a–d).

The differences in the phonon‐electron and phonon‐hole scattering rates at the same concentration are mainly due to variations in the electron DOS within the Fermi window. It should be noted that the magnitude of phonon‐electron matrix elements (|*g*|) for *n*‐type wurtzite semiconductors is always greater than that for *p*‐type wurtzite semiconductors [Figure S6, Supporting Information]. Therefore, the significant impact of EPI on the κ_lat_ in *p*‐type wurtzite semiconductors cannot be explained by |*g*|. **Figure** **4**a,b show that the hole DOS in GaN and ZnO is significantly larger than that of electrons at the carrier concentration of 10^21^ cm^−3^, exhibiting a pronounced asymmetric profile. Furthermore, the X‐ray Photoelectron Spectroscopy (XPS) spectrum reveals that the vicinity of the VBM (0‐5 eV) in GaN is predominantly contributed by Ga4p‐N2p hybridized states,^[^ ^59^ ^]^ which is in agreement with the electronic DOS from our DFT calculations in the corresponding energy region shown in Figure 4a. For typical semiconductors, the states near the band edges exhibit behavior similar to the *s* orbital and the three *p* orbitals observed in individual atoms.^[^ ^60^ ^]^ Given that the *s* orbital predominantly contributes to the DOS, the electron band is more dispersive, resulting in a lower DOS. Conversely, the dominance of the *p*‐orbital leads to minimal band dispersion, thereby resulting in a higher DOS.^[^ ^61^, ^62^, ^63^ ^]^ It is found that the *p*‐orbital components near the Fermi level with the hole concentration of 10^21^ cm^−3^ are significantly higher compared to those for the electron concentration of 10^21^ cm^−3^ for GaN and ZnO, as shown in Figure 4a,b. Therefore, EPI has weak effects on the κ_lat_ of *n*‐type semiconductors, while they have strong effects on that of *p*‐type semiconductors. For AlN, the DOS between the CBM and sub‐conduction band is primarily dominated by *s* orbital, similar to the case in direct bandgap semiconductors. However, the DOS is primarily dominated by *p* orbital above the sub‐conduction band (4.77 eV), leading to a significant increase in DOS [inset picture in Figure 4c]. Therefore, there is a noticeable decrease in κ_lat_ of *n*‐type AlN at the concentration of 10^21^ cm^−3^. For GaP, since the sub‐conduction band (1.40 eV) is relatively close to the CBM, the κ_lat_ of *n*‐type GaP decreases significantly at a concentration of 10^20^ cm^−3^.

### 2.2 Indirect Bandgap Wurtzite Semiconductors

**Figure 5:**
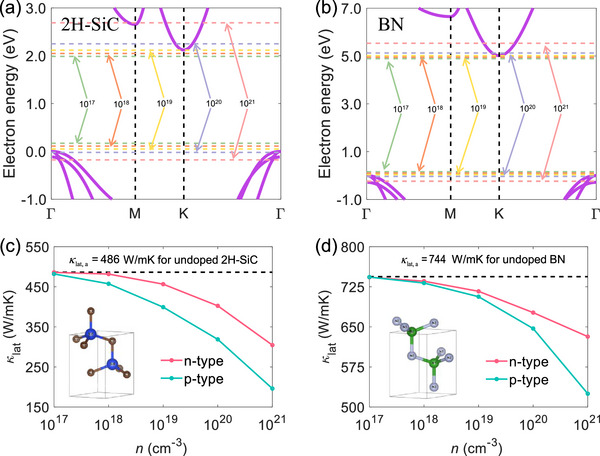
Band structures of a) 2H‐SiC and b) BN along the high symmetry paths, respectively. The horizontal lines are Fermi energy related to the carrier concentrations of 10^17^ (green), 10^18^ (orange), 10^19^ (yellow), 10^20^ (light purple), and 10^21^ (light pink) cm*^−^* ^3^ at room temperature. The electron energy is normalized to the VBM. The κ_lat, *a*_ as a function of carrier concentration at room temperature for undoped, *n*‐type, and *p*‐type c) 2H‐SiC and d) BN, respectively. The crystal structure schematics are inserted at the bottom of the (c) and (d).

**Figure** **5**a,b show the electron band structures of indirect bandgap wurtzite semiconductors along the high‐symmetry path Γ‐M‐K‐Γ in the first Brillouin zone. The VBM is located at the Γ‐point, and the CBM is located at the K‐point, leading to an indirect bandgap of 2.12 and 5.02 eV in 2H‐SiC and BN, respectively.

The variation of κ_lat, *a*_ with carrier concentration is shown in Figure 5c,d. At room temperature, the κ_lat, *a*_ for undoped 2H‐SiC and BN are 486 and 744 W mK^−1^, respectively. Our calculated results are in good agreement with the experimental data^[^ ^64^ ^]^ and former theoretical results.^[^ ^44^, ^65^ ^]^ When the carrier concentration is located in the range of 10^17^ to 10^21^ cm^−3^, a significant decrease in κ_lat, *a*_ is observed in both *n*‐type and *p*‐type 2H‐SiC, with values dropping from 486 to 305 W mK^−1^ and from 482 to 196 W mK^−1^, respectively. Similarly, the κ_lat, *a*_ of *n*‐type BN decreased from 744 to 632 W mK^−1^, while for *p*‐type BN, it dropped from 744 to 525 W mK^−1^. Therefore, the effects of EPI induced by holes and electrons on κ_lat_ in indirect bandgap wurtzite semiconductors are comparable, which is similar to the observation in *fcc* semiconductors.^[^ ^37^, ^39^, ^40^ ^]^

**Figure 6:**
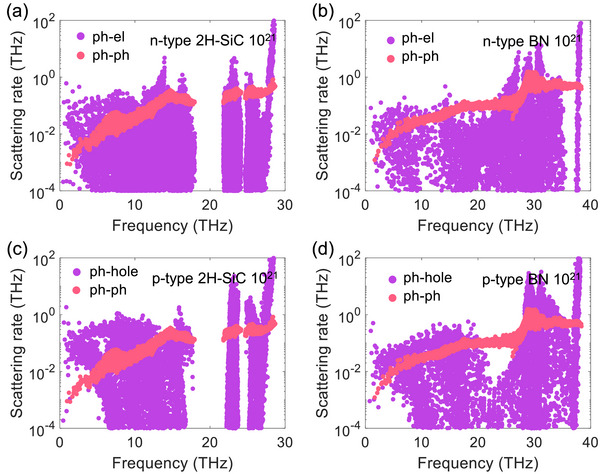
Phonon‐phonon scattering rates, a,b) phonon‐electron scattering rates, and c,d) phonon‐hole scattering rates for 2H‐SiC and BN at room temperature with the carrier concentration of 10^21^ cm*^−^* ^3^.

The phonon‐phonon, the phonon‐electron, and the phonon‐hole scattering rates at room temperature are shown in **Figure** **6**. The phonon‐electron and phonon‐hole scattering rates are calculated at the carrier concentration of 10^21^ cm^−3^. Although the magnitudes of the phonon‐phonon scattering rates for long‐wavelength modes of 2H‐SiC and BN are close, the phonon group velocity of BN is much larger than that of 2H‐SiC, as indicated by their phonon dispersions shown in [Figure S1, Supporting Information]. The phonon‐hole scattering rate is relatively larger than the phonon‐electron scattering rate, resulting in a larger reduction in the *p*‐type 2H‐SiC and BN shown in Figure 5c,d. The magnitudes of both phonon‐electron and phonon‐hole scattering rates of 2H‐SiC are larger than those of BN, which induces the larger relative reduction in κ_lat_ of 2H‐SiC. The phonon‐electron scattering rates at 10^21^cm^−3^ in 2H‐SiC and BN are relatively larger than those in GaN and ZnO, which leads to the significantly different variations in the κ_lat_ with the electron concentration.

**Figure 7:**
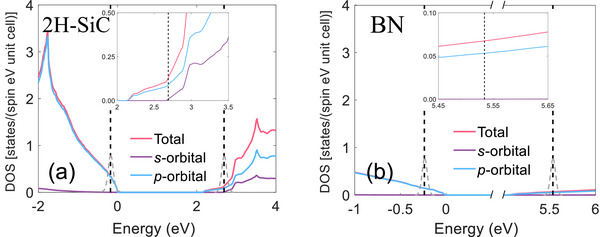
a) Total and partial DOS near the valence‐ and conduction‐band edges of (a) 2H‐SiC and b) BN. The gray dotted curve represents the Fermi window, indicating the relevant range of energies. The electron energy is normalized to the VBM. The position of the Fermi energy for electron and hole concentrations of 10^21^ cm*^−^* ^3^ is indicated with black dashed lines. The enlarged pictures of total and partial DOS for the Fermi energy with the electron concentration of 10^21^ cm*^−^* ^3^ are inserted above in (a,b).

According to the atomic orbital theory, the conduction band component at the Γ‐point of direct bandgap semiconductors is an *s* orbital, while the valence band component is a linear combination of *p* orbitals. However, there is some amount of *p* orbital mixed into *s* orbital in the conduction band of indirect bandgap semiconductors.^[^ ^60^ ^]^ That's why the *p*‐orbital components of 2H‐SiC and BN are higher than those of GaN and ZnO, as shown in the inset picture in **Figure** **7**a,b and Figure 4a,b. As a result, the impact of EPI induced by electrons on κ_lat_ is stronger in indirect bandgap wurtzite semiconductors compared to direct bandgap wurtzite semiconductors.

According to Equation 6 and its variant derived from the double delta approximation,^[^ ^8^, ^9^, ^66^ ^]^ the electron DOS largely determines the Fermi surface nesting function (ζ_***q***_), which can be expressed as:

(1) $$\zeta_{q}=\sum \delta \;\left(\varepsilon_{nk}-\varepsilon_{F}\right)\delta \left(\varepsilon_{mk+q}-\varepsilon_{F}\right)$$

**Figure 8:**
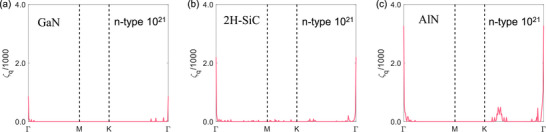
The Fermi surface nesting functions for a) GaN, b) 2H‐SiC, and c) AlN at room temperature, respectively. The carrier concentration is 10^21^ cm*^−^* ^3^ in all cases.

ζ_***q***_ quantifies the phonon‐electron scattering channels.^[^ ^67^ ^]^ As shown in **Figure** **8**, ζ_***q***_ of *n*‐type GaN is much lower than that of *n*‐type 2H‐SiC, which results in its relatively lower phonon‐electron scattering rates. Note that there is an additional scattering channel generated by the sub‐conduction band located at the K‐point, which is 0.73 eV away from the CBM in AlN as the electron concentration increases from 10^20^ to 10^21^ cm^−3^, as shown in [Figure S7, Supporting Information]. Therefore, there is a peak in the ζ_***q***_ in the vicinity of the K‐point. Although ζ_***q***_ of *n*‐type AlN is much larger than that of *n*‐type 2H‐SiC, the phonon linewidth ($\Gamma_{\mathrm{pe}}=1/2\tau_{\lambda }^{\mathrm{ph}-\mathrm{el}}$) of *n*‐type AlN is significantly smaller than that of *n*‐type 2H‐SiC [Figure S8, Supporting Information]. Therefore, the stronger EPI effects on κ_lat_ of 2H‐SiC cannot be interpreted by ζ_***q***_.

**Figure 9:**
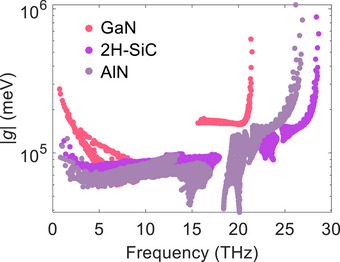
Absolute value of electron‐phonon matrix elements |*g*| with respect to the phonon frequency of GaN, 2H‐SiC, and AlN.

Note that the phonon‐electron scattering rate is also related to the electron‐phonon coupling strength *g*. The electron‐phonon coupling elements *g* quantifies the coupling strength between phonon modes and electron states,

(2) $$g_{mn}^{\nu }\left(k,q\right)=\sqrt{\frac{\hbar }{2\omega_{\lambda }}}\;\langle \psi_{mk+q}\left|\partial_{\lambda }V\right|\psi_{nk}\rangle$$

with ψ the ground‐state Bloch wave function and ∂_λ_ *V* the first‐order derivative of the Kohn‐Sham potential with respect to the atomic displacement. The phonon‐electron scattering rate has a positive relationship with ζ_***q***_ and |*g*|. As shown in **Figure** **9**, the magnitude of |*g*| for 2H‐SiC is larger than that of AlN, resulting in the smaller κ_lat_ for 2H‐SiC. Furthermore, although GaN has the largest magnitude of |*g*|, its much smaller ζ_***q***_ leads to weaker effects of EPI on κ_lat_.

## 3 Conclusion 

In summary, we perform a comprehensive investigation on the phonon thermal transport in direct/indirect bandgap wurtzite semiconductors, incorporating the phonon‐phonon, phonon‐electron, and phonon‐isotope scatterings. Overall, the EPI has a larger effect on the lattice thermal conductivity of *p*‐type doping compared to *n*‐type doping in the same wurtzite semiconductor. This is attributed to the relatively higher electron density of states within the Fermi window caused by *p* orbitals, which significantly increases the phonon‐electron scattering channels. For *n*‐type doping, the EPI exhibits a significantly stronger influence on the lattice thermal conductivity of indirect bandgap semiconductors compared to direct bandgap semiconductors. This is attributed to the relatively lower electron density of states in direct bandgap semiconductors within the Fermi window stemming from the *s*‐orbital component. Nonetheless, the dramatic reduction in lattice thermal conductivity in the direct bandgap semiconductors AlN and GaP at high electron concentrations is attributed to additional phonon‐electron scattering channels induced by the sub‐conduction bands. Moreover, it is the electron‐phonon matrix element, rather than the Fermi surface nesting function, that is ascribed to the relatively larger reduction in the lattice thermal conductivity of *n*‐type 2H‐SiC compared to *n*‐type AlN at ultrahigh concentrations.

## 4 Simulation Section

Combining the linearized phonon Boltzmann transport equation and Fourier's law, the lattice thermal conductivity (κ_lat_) can be calculated as:^[^ ^68^ ^]^

(3) $$\kappa_{\mathrm{lat},\alpha \beta }=\sum_{\lambda }c_{\nu ,\lambda }\;v_{\lambda ,\alpha }v_{\lambda ,\beta }\tau_{\lambda }$$

where α and β are the Cartesian coordinates, λ ≡ (***q***, ν) denotes the phonon mode with wave vector ***q*** and phonon polarization ν. The phonon specific heat capacity is denoted as $c_{\nu ,\lambda }=\frac{1}{V}\;\hbar \omega_{\lambda }\frac{\partial n_{\lambda }}{\partial T}$, where *V* is the volume of the primitive cell, ω_λ_ is the phonon frequency, *n*_λ_ is the Bose‐Einstein distribution at temperature *T. v*_λ_ is the phonon group velocity, and τ_λ_ is the phonon relaxation time, which can be obtained using Matthiessen's rule as $1/\;\tau_{\lambda }=1/\tau_{\lambda }^{\mathrm{ph}-\mathrm{ph}}+1/\tau_{\lambda }^{\mathrm{ph}-\mathrm{iso}}+1/\tau_{\lambda }^{\mathrm{ph}-\mathrm{el}}$, where $1/\tau_{\lambda }^{\mathrm{ph}-\mathrm{ph}}$ is the phonon‐phonon scattering rate, $1/\tau_{\lambda }^{\mathrm{ph}-\mathrm{iso}}$ is the phonon‐isotope scattering rate, and $1/\tau_{\lambda }^{\mathrm{ph}-\mathrm{el}}$ is the phonon‐electron scattering rate.

The phonon‐phonon scattering rate due to three‐phonon scattering is given by Fermi's golden rule as:^[^ ^69^ ^]^

(4) $$\begin{array}{cc} \frac{1}{\tau_{\lambda }^{\mathrm{ph}-\mathrm{ph}}} & =2\pi \sum_{\lambda_{1}\lambda_{2}}{\left|V_{\lambda \lambda_{1}\lambda_{2}}\right|}^{2}\\ & \times \left[\frac{1}{2}\left(1+n_{\lambda_{1}}^{0}+n_{\lambda_{2}}^{0}\right)\delta \left(\omega_{\lambda }-\omega_{\lambda_{1}}-\omega_{\lambda_{2}}\right)\right.\\ & +\left.\left(n_{\lambda_{1}}^{0}-n_{\lambda_{2}}^{0}\right)\delta \left(\omega_{\lambda }+\omega_{\lambda_{1}}-\omega_{\lambda_{2}}\right)\right] \end{array}$$

$V_{\lambda \lambda_{1}\lambda_{2}}$ denotes the three‐phonon scattering matrix element, which is related to the cubic force constants. δ is the Dirac delta function, which ensures the conservation of energy during the scattering processes.

The phonon‐isotope scattering was treated under the assumption of a random distribution of isotopes as:^[^ ^70^ ^]^

(5) $$\frac{1}{\tau_{\lambda }^{\mathrm{iso}}}=\frac{\pi \omega_{\lambda }^{2}}{2N}\;\sum_{i\in u.c.}g\left(i\right){\left|e_{\lambda^{\prime }}^{*}\cdot e_{\lambda }\left(i\right)\right|}^{2}\delta \left(\omega_{\lambda }-\omega_{\lambda^{\prime }}\right)$$

where ***e*** is the normalized eigenvector of phonon mode λ and the asterisk denotes the complex conjugate, $g\left(i\right)=\sum_{i}n_{i}\;\left(j\right){\left[1-m_{i}\left(j\right)/{\bar{m}}_{i}\left(j\right)\right]}^{2}$, where *n_i_* (*j*) and *m_i_* (*j*) are the concentration and atomic mass of the *i*th substitution atom, respectively. ${\bar{m}}_{i}\left(j\right)$ is the average mass of the *j*th atom in the unit cell.

The phonon‐electron scattering rate is related to the imaginary part of the phonon self‐energy, which can be expressed as:^[^ ^71^ ^]^

(6) $$\begin{array}{cc} & \frac{1}{\tau_{\lambda }^{\mathrm{ph}-\mathrm{el}}}\\ & \;=-\frac{2\pi }{\hbar }\sum_{mn,k}{\left|g_{mn}^{\nu }\left(k,q\right)\right|}^{2}\times \left(f_{nk}-f_{mk+q}\right)\delta \left(\varepsilon_{mk+q}-\varepsilon_{nq}-\hbar \omega_{\lambda }\right) \end{array}$$

where *g*_*mn*,ν_(***k***, ***q***) is the electron‐phonon matrix element, which quantifies the probability amplitude for scattering between the electronic state *n**k*** and *m**k*** + ***q***. The Fermi‐Dirac distribution function is denoted as*f*(ε)  =  1/exp ((ε − ε_*F*_)/*k_B_T*) + 1, where *k_B_* is the Boltzmann constant, ε is the electronic band energy, and ε_*F*_ is the Fermi energy.

The first‐principles calculations are performed using the Quantum Espresso package.^[^ ^72^ ^]^ The electron exchange‐correlation functional is treated by the generalized gradient approximation of Perdew–Burke‐ Ernzerhof (PBE)^[^ ^73^ ^]^ and optimized full relativistic norm‐conserving pseudopotentials^[^ ^74^ ^]^ from PseudoDojo.^[^ ^75^ ^]^ The lattice vectors and atomic positions are fully relaxed based on the Broyden–Fretcher–Goldfarb–Shanno (BFGS) optimization method^[^ ^76^, ^77^, ^78^, ^79^ ^]^ and the convergence thresholds for energy and force are set to 10^−8^ Ry and 10^−8^ Ry/Bohr. The kinetic energy cutoff for plane waves is set to be 120 Ry. The Brillouin zone is sampled with a 12×12×8 Monkhorst‐Pack **k**‐point mesh to ensure convergence for self‐consistent calculation. The threshold of electron total energy is set to be 10^−10^ Ry. The lattice constants of the materials in our work agree well with the experimental data,^[^ ^80^, ^81^ ^]^ as shown in [Table S3, Supporting Information]. The harmonic force constants are calculated from density‐functional perturbation theory (DFPT).^[^ ^82^ ^]^ The **q**‐point mesh is set to be 6 × 6 × 4 and the energy threshold is 10^−17^ Ry to guarantee convergence. Also, the dielectric constant and Born effective charge are calculated to account for the long‐range electrostatic interactions. The phonon dispersions are in good agreement with the experimental data,^[^ ^83^, ^84^ ^]^ as shown in [Figure S1, Supporting Information]. The cubic force constants are also calculated based on DFPT.^[^ ^85^ ^]^ The **q**‐point mesh is set to be 3 × 3 × 2. The phonon‐electron scattering rates and the magnitude of phonon‐electron matrix elements are calculated by our in‐house modified Electron‐Phonon Wannier package.^[^ ^71^ ^]^ The electron band structures calculated by density functional theory agree quite well with those obtained through the Wannier technique, as shown in [Figure S2, Supporting Information]. The convergence of the phonon‐electron scattering rates with respect to the **k**‐point mesh is presented in [Figure S3 and S4, Supporting Information]. The phonon‐electron scattering rates exhibit minor variations when using **k**‐point meshes of 24 × 24 × 16, 36 × 36 × 24, and 45 × 45 × 30. Therefore, we set the **k**‐point mesh to 36 × 36 × 24 for the EPI calculations of all wurtzite semiconductors. The electron‐phonon coupling matrix elements are first calculated under coarse **k** */* **q**‐point meshes (12 × 12 × 8/6 × 6 × 4), and then interpolated to dense **k** */* **q**‐point meshes (36×36×24/21×21×14) with the Wannier interpolation technique.^[^ ^86^ ^]^ The in‐house modified D3Q package^[^ ^85^ ^]^ is employed to calculate the κ_lat_, incorporating the phonon‐electron scattering rate using the iterative calculation scheme.^[^ ^7^, ^9^, ^42^ ^]^ This framework has been rigorously validated in our recent works for its accuracy.^[^ ^8^, ^42^ ^]^ κ_lat_ is converged with respect to a **q**‐point mesh of 21 × 21 × 14. The carrier concentration of 10^21^ cm^−3^ can be experimentally realized in bulk semiconductors.^[^ ^87^, ^88^ ^]^ Therefore, we limited the charge carrier concentration range from 10^17^ to 10^21^ cm^−3^. The rigid shift of the Fermi energy is utilized to imitate the change of carrier concentration.^[^ ^29^, ^89^ ^]^

## Conflict of Interest

The authors declare no conflict of interest.

## Supporting information



Supporting Information

## Data Availability

The data that support the findings of this study are available from the corresponding author upon reasonable request.
