# Supplementary material for: Diverse Responses in Lattice Thermal Conductivity of n‐Type/p‐Type Wurtzite Semiconductors Driven by Asymmetric Electron‐Phonon Interactions
Source: Adv Sci (Weinh). 2025 Oct 27;13(2):e14910. doi: 10.1002/advs.202514910 (PMC12786365; doi:10.1002/advs.202514910)
Supplement: Supplementary file 1 — Supporting Information [file ADVS-13-e14910-s001.pdf]

Diverse Responses in Lattice Thermal Conductivity of n-type/p-type Wurtzite Semiconductors Driven by Asymmetric Electron-Phonon Interactions

Supporting Information

Jianshi Sun,[1] Shouhang Li,[2,*] Zhen Tong,[3] Cheng Shao,[4] Han Xie,[5] Meng An,[6] Chuang Zhang,[1] Xiongfei Zhu,[1] Chen Huang,[1] Quanjie Wang,[7] Yucheng Xiong,[1] and Xiangjun Liu[1,†]

[1]*Institute of Micro/Nano Electromechanical System and Integrated Circuit, College of Mechanical Engineering, Donghua University, Shanghai 201620, China*

[2]*Centre de Nanosciences et de Nanotechnologies, CNRS, Université Paris-Saclay, 10 Boulevard Thomas Gobert, Palaiseau 91120, France*

[3]*School of Advanced Energy, Sun Yat-Sen University, Shenzhen 518107, China*

[4]*Thermal Science Research Center, Shandong Institute of Advanced Technology, Jinan, Shandong 250103, China*

[5]*School of Energy and Materials, Shanghai Polytechnic University, Shanghai 201209, China*

[6]*Department of Mechanical Engineering, The University of Tokyo, 7-3-1 Hongo, Bunkyo, Tokyo, 113-8656, Japan*

[7]*School of Mechanical Engineering, Shandong Key Laboratory of CNC Machine Tool Functional Components, Qilu University of Technology (Shandong Academy of Sciences), Jinan 250353, China*

(Dated: October 1, 2025)

* shouhang.li@universite-paris-saclay.fr
† xjliu@dhu.edu.cn

I. Phonon dispersions

As shown in Fig. S1, the phonon dispersions of all materials exhibit no negative frequencies, indicating that they are thermodynamically stable. Moreover, the phonon frequencies of GaN, ZnO, and AlN agree well with experimental data[17, 20, 21].

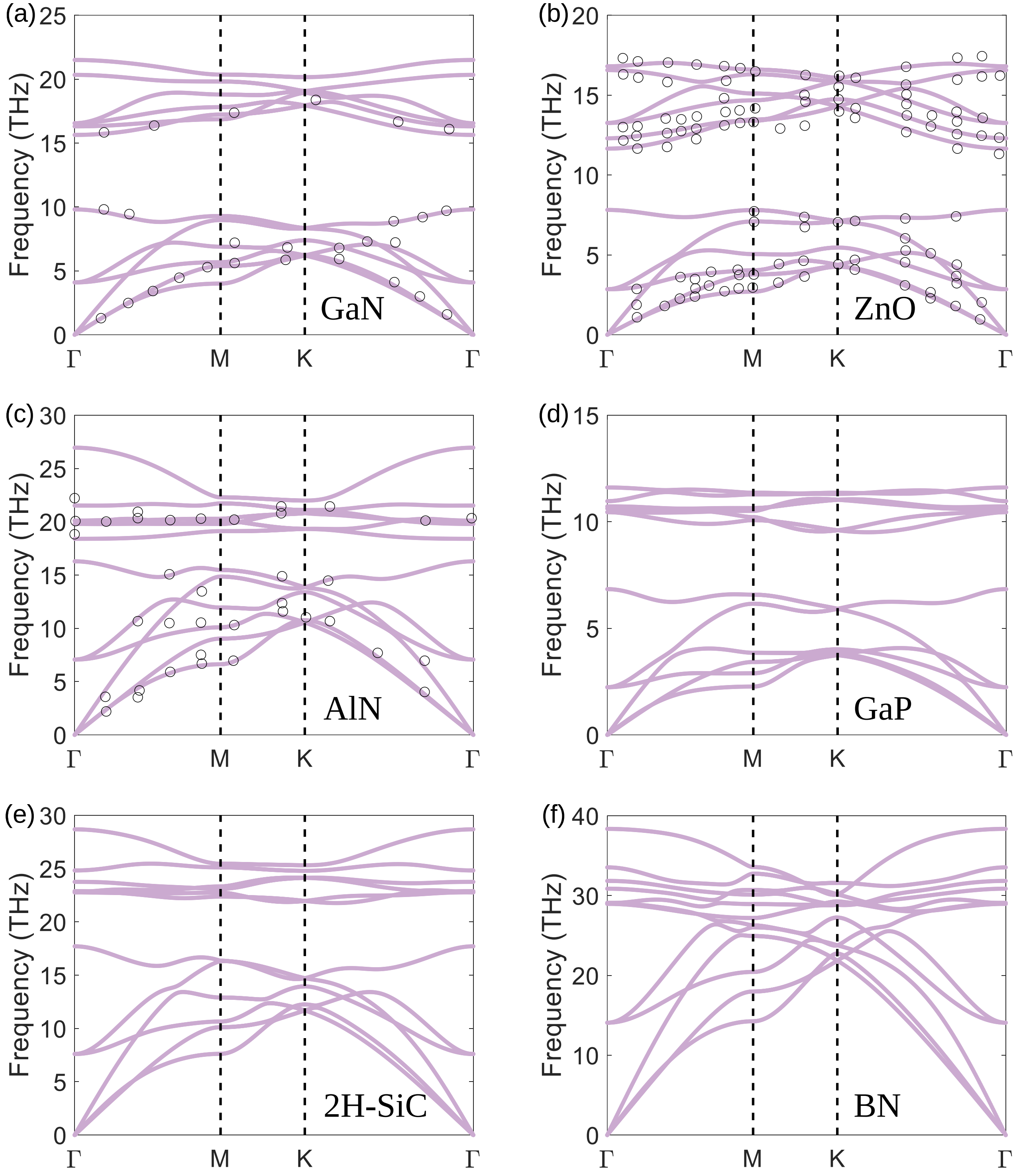

FIG. S1. Phonon dispersions of (a) GaN, (b) ZnO, (c) AlN, (d) GaP, (e) 2H-SiC, and (f) BN, respectively. The experimental data (symbols) are taken from Ref.[17, 20, 21].

II. The validation of wannierization

We adopt the selected columns of the density matrix method (SCDM)[4, 5] to automatically generate maximally-localized Wannier functions. Fig. S2 shows the electronic band structures calculated by DFT and Wannier interpolation. The results match quite well with each other, indicating the reliability of Wannierization.

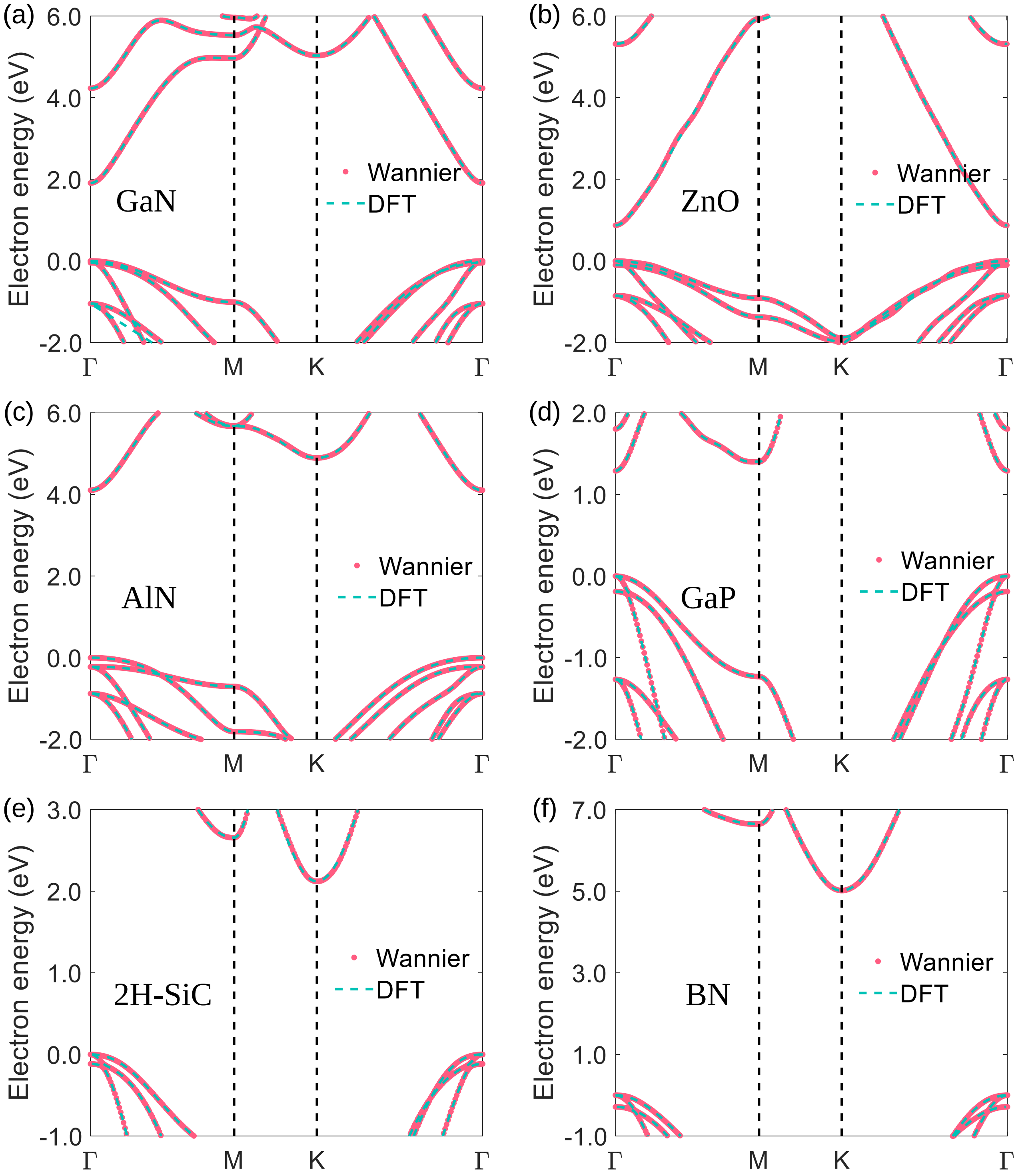

FIG. S2. The electronic band structures calculated by DFT and Wannier interpolation for (a) GaN, (b) ZnO, (c) AlN, (d) GaP, (e) 2H-SiC, and (f) BN, respectively. The electron energy is normalized to the valence band maximum.

III. The convergence tests on k-point mesh

In Figs. S3 and S4, the phonon-electron scattering rates of n-type and p-type wurtzite semiconductors are shown for **k**-point meshes of $24 \times 24 \times 16$, $36 \times 36 \times 24$, and $45 \times 45 \times 30$. It can be seen that the phonon-electron scattering rates do not have significant variations using these **k**-point meshes. Therefore, we adopt $36 \times 36 \times 24$ **k**-point mesh in our calculations.

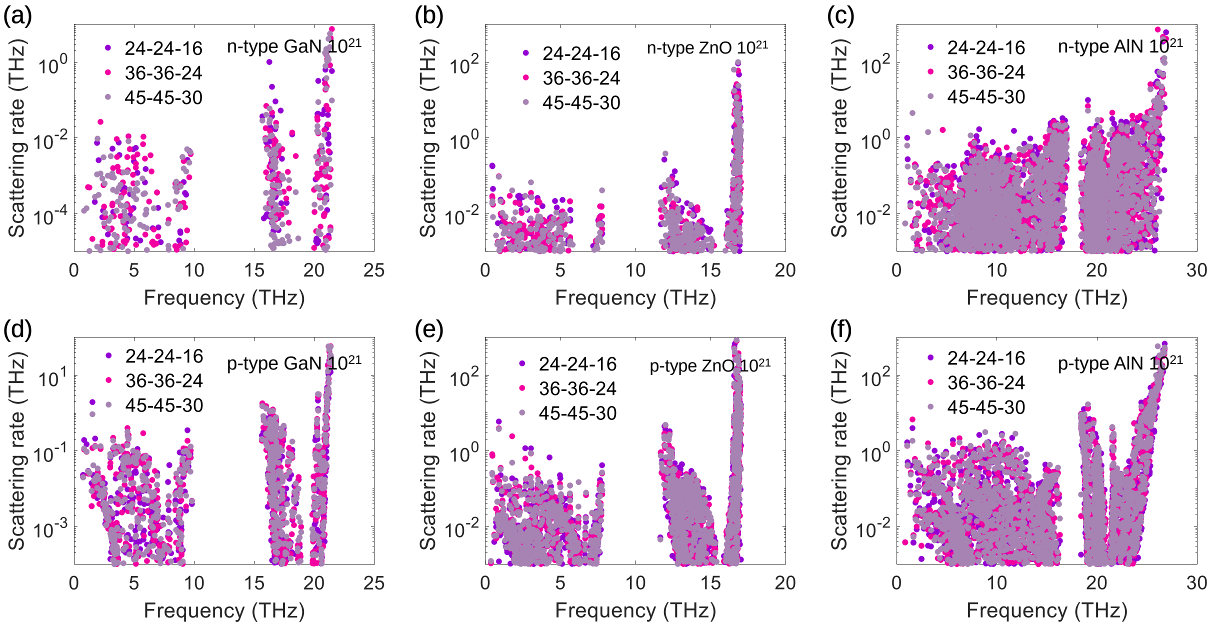

FIG. S3. Phonon-electron scattering rates of (a-c) n-type and (d-f) p-type of GaN, ZnO, and AlN versus the different **k**-point meshes. The carrier concentration is 10^{21} cm^{-3} in all the cases.

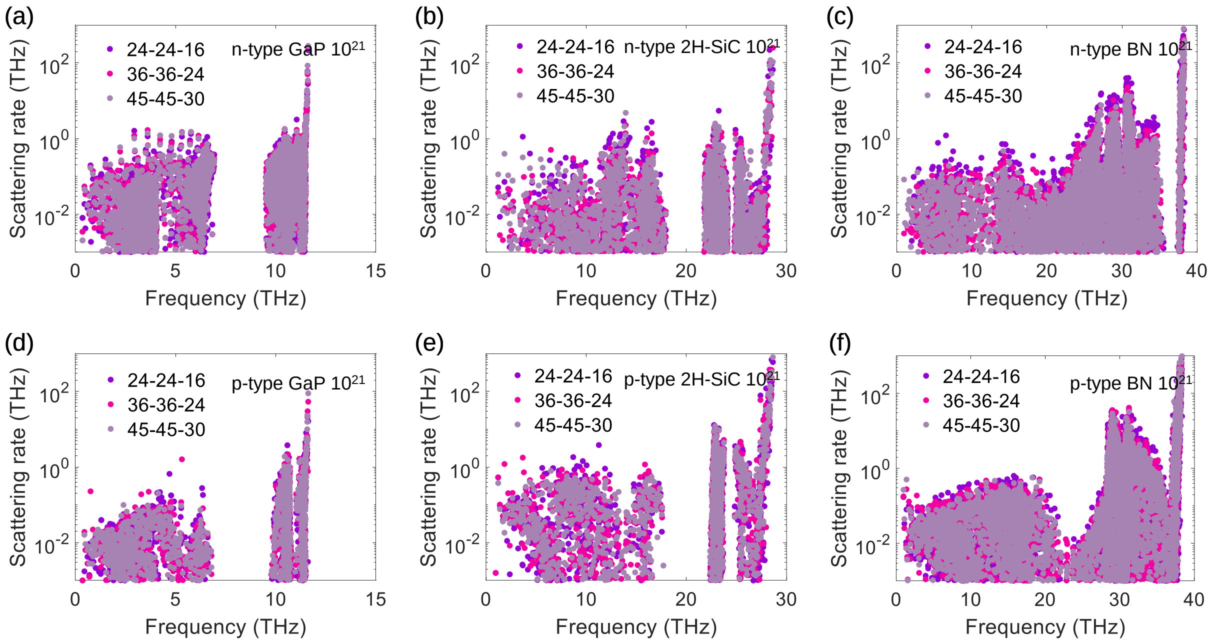

FIG. S4. Phonon-electron scattering rates of (a-c) n-type and (d-f) p-type of GaP, 2H-SiC, and BN versus the different **k**-point meshes. The carrier concentration is 10^{21} cm^{-3} in all the cases.

IV. Phonon-electron and phonon-hole scattering rates in GaN and AlN at $10^{20}\,\mathrm{cm}^{-3}$ carrier concentration

Fig. S5 shows the trends of phonon-electron and phonon-hole scattering rates in AlN resemble those in GaN when the carrier concentration reaches $10^{20}\,\mathrm{cm}^{-3}$.

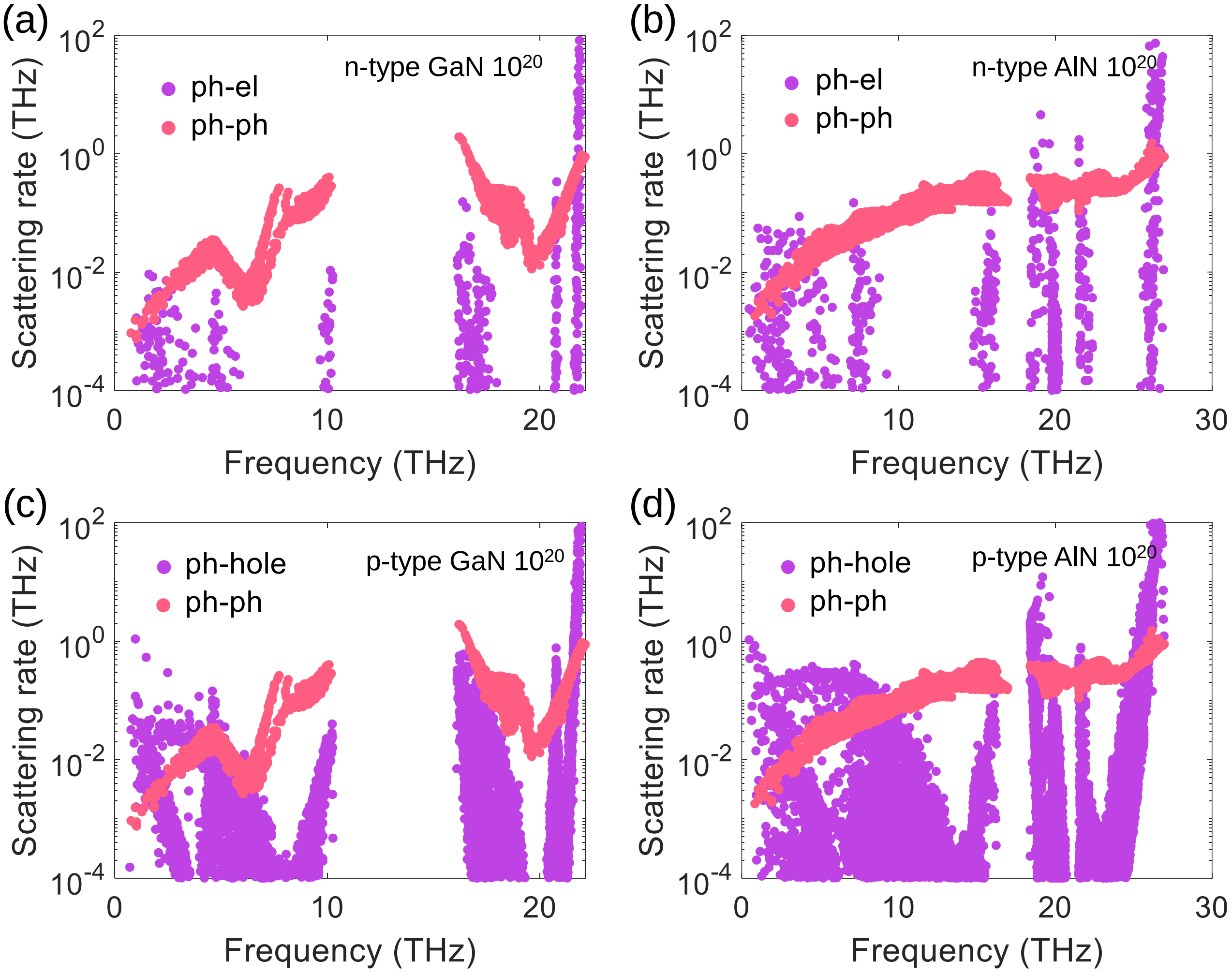

FIG. S5. Phonon-phonon scattering rates, (a-b) phonon-electron scattering rates, and (c-d) phonon-hole scattering rates for GaN and AlN at room temperature with carrier concentration of $10^{20}\,\mathrm{cm}^{-3}$.

V. Absolute value of electron-phonon matrix elements $|g|$

Fig. S6 shows the magnitude of phonon-electron matrix elements ($|g|$) for n-type wurtzite semiconductors is always greater than that for p-type wurtzite semiconductors. Therefore, the significant impact of EPI on the κ_{lat} in p-type cannot be explained by $|g|$.

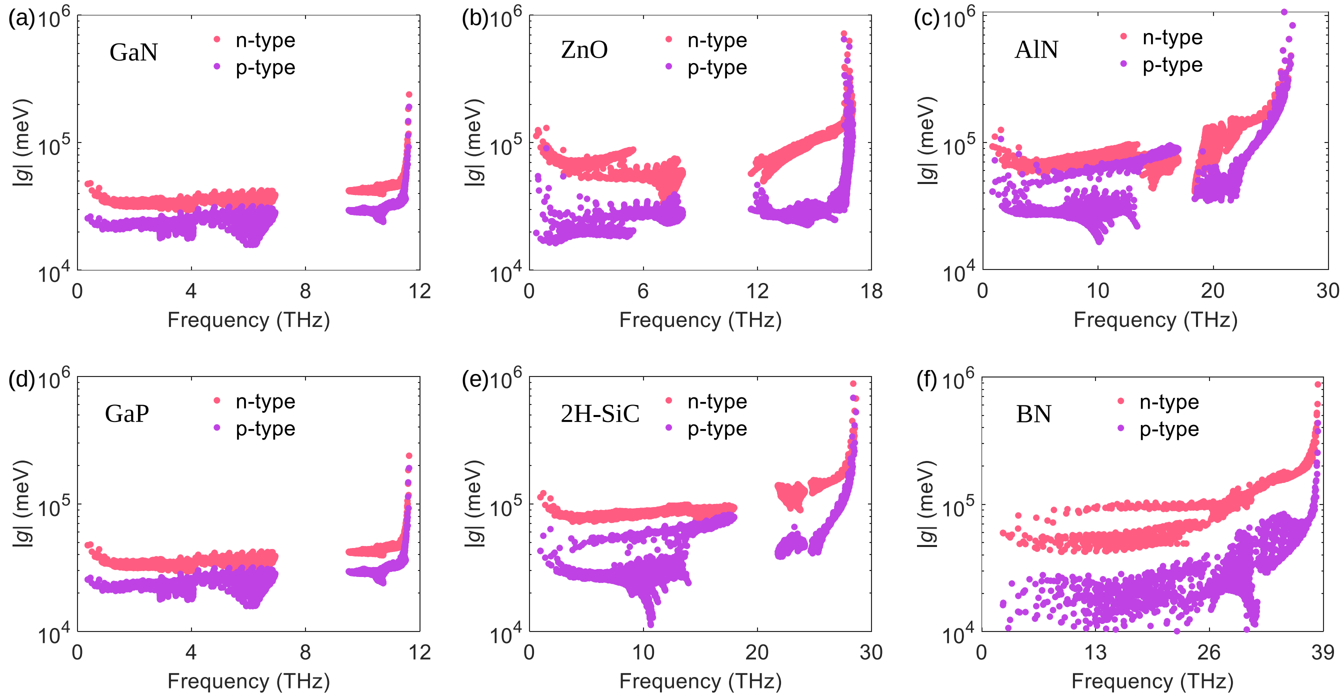

FIG. S6. Absolute value of electron-phonon matrix elements $|g|$ with respect to the phonon frequency.

VI. Fermi surface nesting functions for AlN

As shown in Fig. S7, an additional scattering channel appears in the Fermi surface nesting functions of n-type AlN, leading to an anomalous decrease in the lattice thermal conductivity as the carrier concentration ranges from 10^{20} to 10^{21} cm^{-3}.

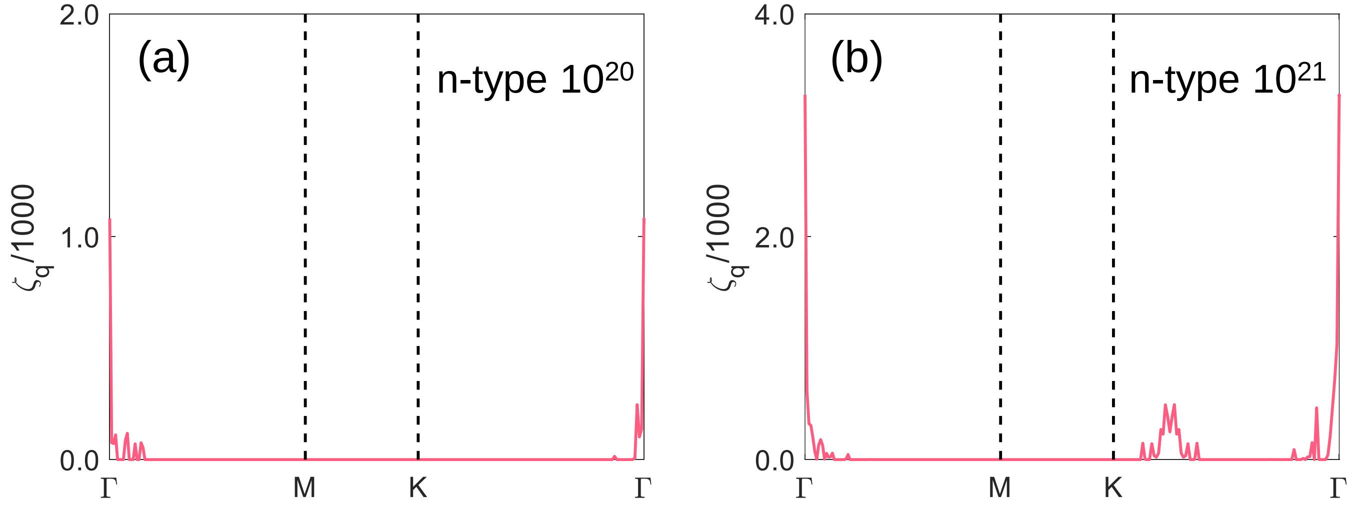

FIG. S7. The Fermi surface nesting functions for n-type AlN at room temperature. The carrier concentrations are (a) 10^{20} and (b) 10^{21} cm^{-3}.

VII. Phonon linewidths for 2H-SiC and AlN

As shown in Fig. S8, the phonon linewidth of n-type 2H-SiC is larger than that of n-type AlN. Therefore, the relatively large reduction in κ_{lat} of n-type 2H-SiC cannot be explained by the Fermi surface nesting functions and should be ascribed to the electron-phonon matrix elecments.

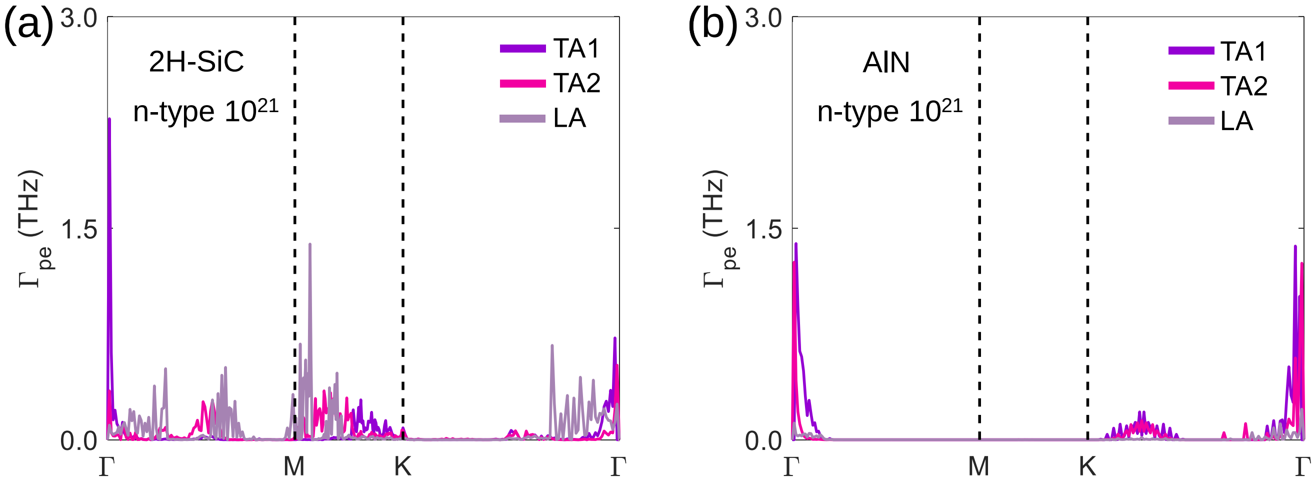

FIG. S8. Room temperature phonon linewidth Γ_{pe} of TA1, TA2, and LA along high-symmetry path due to phonon-electron scattering with electron concentration of 10^{21} cm^{-3} for (a) 2H-SiC and (b) AlN.

VIII. Verify first-principles calculations

Table S1 shows the lattice thermal conductivity of undoped GaN calculated using the Perdew-Burke-Ernzerhof (PBE) form of the exchange-correlation functional[14] with fully relativistic Optimized Norm-Conserving Vanderbilt (ONCV)[8], Projector-Augmented Wave (PAW)[3], and ultrasoft (US) pseudopotentials[10]. It is found that the lattice thermal conductivity is insensitive to the choice of pseudopotentials. In addition, the lattice constants calculated using the PBE pseudopotential with fully relativistic ONCV in this work show better agreement with the experimental data[18]. Therefore, we adopt this type of pseudopotential for all cases.

TABLE S1. The lattice constants (a and c) and $\kappa_{\text{lat},a}$ of undoped GaN obtained from first-principles calculations using different pseudopotentials and experiment.

	ONCV	PAW	US	Experiment
a (Å)	3.184	3.179	3.204	3.190[18]
c (Å)	5.187	5.171	5.206	5.189[18]
$\kappa_{\text{lat},a}$ (W/mK)	264	245	278	269[9]

IX. Mode-level lattice thermal conductivity of GaN

As shown in Table S2, the $\kappa_{\text{lat},a}$ of undoped GaN is dominated by acoustic phonons and mid-frequency optical phonons, while the high-frequency optical phonons have marginal contributions. With phonon-electron scattering incorporated, the contributions of acoustic phonons in n-type GaN decrease, while mid-frequency optical phonons have dominant contributions. This trend is further strengthened in p-type GaN.

TABLE S2. The contributions of $\kappa_{\text{lat},a}$ in undoped and n-type/p-type GaN from acoustic phonons, mid-frequency optical phonons, and high-frequency optical phonons, respectively.

GaN	Acoustic phonons	Mid-frequency optical phonons	High-frequency optical phonons
undoped	49%	45%	6%
n-type (10^{21} cm^{-3})	41%	55%	4%
p-type (10^{21} cm^{-3})	33%	60%	7%

X. First-principles calculations results and experimental data

TABLE S3. The band gap, lattice constants (a and c), and $\kappa_{\mathrm{lat},a}$ of wurtzite semiconductors obtained from experimental data and first-principles calculations.

		GaN	ZnO	AlN	GaP	2H-SiC	BN
Band gap (eV)	This work	1.86	0.87	4.11	1.29	2.12	5.02
	Experiment	3.47[11]	3.30[13]	6.03[7]	2.26[1]	3.33[12]	5.50[11]
a (Å)	LDA	3.178	3.186	3.057	3.784	3.011	2.495
	PBE	3.184	3.225	3.108	3.833	3.076	2.542
	PBEsol	3.195	3.336	3.255	3.899	3.154	2.611
	Experiment	3.190[18]	3.253[19]	3.110[18]	3.842[2]	3.079[19]	2.553[22]
c (Å)	LDA	5.142	5.098	4.888	6.125	4.989	4.158
	PBE	5.187	5.181	4.963	6.320	5.048	4.205
	PBEsol	5.244	5.366	5.121	6.444	5.201	4.333
	Experiment	5.189[18]	5.231[19]	4.980[18]	6.335[2]	5.053[19]	4.228[22]
$\kappa_{\mathrm{lat},a}$ (W/mK)	Undoped (This work)	264	37	295	82	486	744
	Undoped (Previous work)	269[9]	45[6]	317[24]	97[23]	497[15]	907[16]

[1] Ashcroft, N.W., Mermin, N.D., 1976. Solid state. Physics (New York: Holt, Rinehart and Winston) Appendix C 1.
[2] Assali, S., Zardo, I., Plissard, S., Kriegner, D., Verheijen, M., Bauer, G., Meijerink, A., Belabbes, A., Bechstedt, F., Haverkort, J., et al., 2013. Direct band gap wurtzite gallium phosphide nanowires. Nano Letters 13, 1559–1563.
[3] Blöchl, P.E., 1994. Projector augmented-wave method. Physical Review B 50, 17953.
[4] Damle, A., Lin, L., 2018. Disentanglement via entanglement: a unified method for Wannier localization. Multiscale Modeling & Simulation 16, 1392–1410.
[5] Damle, A., Lin, L., Ying, L., 2015. Compressed representation of Kohn–Sham orbitals via selected columns of the density matrix. Journal of Chemical Theory and Computation 11, 1463–1469.
[6] Dash, S., Padhan, P., 2024. Lattice thermal conductivity of zno: experimental and theoretical studies. Physical Chemistry Chemical Physics 26, 14754–14765.
[7] Guo, Q.G.Q., Yoshida, A.Y.A., 1994. Temperature dependence of band gap change in inn and aln. Japanese Journal of Applied Physics 33, 2453.
[8] Hamann, D., 2013. Optimized norm-conserving Vanderbilt pseudopotentials. Physical Review B 88, 085117.
[9] Jeżowski, A., Churiukova, O., Mucha, J., Suski, T., Obukhov, I., Danilchenko, B., 2015. Thermal conductivity of heavily doped bulk crystals GaN: O. Free carriers contribution. Materials Research Express 2, 085902.
[10] Kresse, G., Joubert, D., 1999. From ultrasoft pseudopotentials to the projector augmented-wave method. Physical Review B 59, 1758.
[11] Levinshtein, M.E., Rumyantsev, S.L., Shur, M.S., 2001. Properties of Advanced Semiconductor Materials: GaN, AIN, InN, BN, SiC, SiGe. John Wiley & Sons.
[12] Patrick, L., Hamilton, D., Choyke, W., 1966. Growth, luminescence, selection rules, and lattice sums of sic with wurtzite structure. Physical Review 143, 526.
[13] Pawar, V., Jha, P.K., Panda, S., Jha, P.A., Singh, P., 2018. Band-gap engineering in ZnO thin films: a combined experimental and theoretical study. Physical Review Applied 9, 054001.
[14] Perdew, J.P., Ruzsinszky, A., Csonka, G.I., Vydrov, O.A., Scuseria, G.E., Constantin, L.A., Zhou, X., Burke, K., 2008. Restoring the density-gradient expansion for exchange in solids and surfaces. Physical Review Letters 100, 136406.
[15] Protik, N.H., Katre, A., Lindsay, L., Carrete, J., Mingo, N., Broido, D., 2017. Phonon thermal transport in 2H, 4H and 6H silicon carbide from first principles. Materials Today Physics 1, 31–38.
[16] Raya-Moreno, M., Rurali, R., Cartoixà, X., 2019. Thermal conductivity for iii-v and ii-vi semiconductor wurtzite and zinc-blende polytypes: The role of anharmonicity and phase space. Physical Review Materials 3, 084607.
[17] Ruf, T., Serrano, J., Cardona, M., Pavone, P., Pabst, M., Krisch, M., D'astuto, M., Suski, T., Grzegory, I., Leszczynski, M., 2001. Phonon dispersion curves in wurtzite-structure GaN determined by inelastic x-ray scattering. Physical Review Letters 86, 906.
[18] Schulz, H., Thiemann, K., 1977. Crystal structure refinement of AlN and GaN. Solid State Communications 23, 815–819.
[19] Schulz, H., Thiemann, K., 1979. Structure parameters and polarity of the wurtzite type compounds SiC—2H and ZnO. Solid State Communications 32, 783–785.
[20] Schwoerer-Böhning, M., Macrander, A., Pabst, M., Pavone, P., 1999. Phonons in wurtzite aluminum nitride. Physica Status Solidi (b) 215, 177–180.

[21] Serrano, J., Manjón, F., Romero, A., Ivanov, A., Cardona, M., Lauck, R., Bosak, A., Krisch, M., 2010. Phonon dispersion relations of zinc oxide: Inelastic neutron scattering and ab initio calculations. Physical Review B 81, 174304.
[22] Sōma, T., Sawaoka, A., Saito, S., 1974. Characterization of wurtzite type boron nitride synthesized by shock compression. Materials Research Bulletin 9, 755–762.
[23] Togo, A., Chaput, L., Tanaka, I., 2015. Distributions of phonon lifetimes in Brillouin zones. Physical Review B 91, 094306.
[24] Yuan, K., Zhang, X., Tang, D., Hu, M., 2018. Anomalous pressure effect on the thermal conductivity of ZnO, GaN, and AlN from first-principles calculations. Physical Review B 98, 144303.
